# Warm-Water Footbathing in Young Women With Cold-Sensitivity Constitution (Hiesho) Increases Parasympathetic Nerve Activity and Promotes Peripheral Circulation

**DOI:** 10.7759/cureus.89470

**Published:** 2025-08-06

**Authors:** Kaori Kono, Ryo Kayashima, Shichiro Abe, Toshiaki Nakajima, Shigeru Toyoda

**Affiliations:** 1 Fundamental and Functional Nursing, Faculty of Nursing, Dokkyo Medical University, Mibu, JPN; 2 Department of Cardiovascular Medicine, Dokkyo Medical University, Mibu, JPN

**Keywords:** autonomic nervous activity, cold-sensitivity constitution (csc), footbathing, heart rate variability, parasympathetic nerve activity, peripheral circulation, young women

## Abstract

Background

A cold-sensitivity constitution (CSC), termed “Hiesho” in Japanese, is a common condition among young women that impairs quality of life through reduced peripheral circulation and autonomic imbalance. In our previous study, we reported that cold intolerance is associated with an imbalance in autonomic nervous function, as evaluated by heart rate variability (HRV). Conversely, footbathing increases parasympathetic nervous activity (PNA) and increases both peripheral blood flow and epidermal temperature. In the present study, we aimed to compare the autonomic nervous activity and peripheral skin temperatures of young women with CSC using either warm-water or steam footbathing.

Methods

This study employed a quasi-experimental, cross-sectional design. The participants were 12 healthy young women diagnosed with CSC (six in the warm-water footbathing group and six in the steam footbathing group). All participants reported sensations of coldness, were identified as having CSC via the Coldness Survey Questionnaire, and exhibited contrast between central (tympanic) and peripheral (hallux) body temperature measurements of 6°C. Fifteen minutes before and at 15 and 30 minutes after a 15-minute footbathing, we measured the epidermal temperature at the hallux and dorsal hand. Heart rate variability (HRV) was also assessed.

Results

The parasympathetic activity index tended to increase in the warm-water footbathing group 15 minutes after footbathing, although the difference was not statistically significant (p=0.065). No statistically significant differences were observed in sympathetic nerve activity index or heart rate between the two (warm-water and steam) groups. The right dorsal hand skin temperature showed an increasing trend in the warm-water footbathing group at 15 and 30 minutes after the footbath, although the difference was not statistically significant (p=0.093 at 15 min, p=0.065 at 30 min). Notably, the right hallux skin temperature increased significantly more in the warm-water footbathing group 30 minutes after footbathing (p=0.026).

Conclusion

Warm-water footbathing for healthy young women with CSC increased PNA and peripheral skin temperature more effectively than steam footbathing.

## Introduction

A cold-sensitivity constitution (CSC), known in Japan as “Hiesho,” is estimated to affect nearly half of all women [1]. Although frequently observed in postmenopausal women [2], its prevalence is increasing among women in their twenties [3]. According to recent Japanese epidemiological data, over 50% of young women report symptoms consistent with CSC, suggesting a rising trend that requires preventive intervention [4].

CSC is defined as a physiological condition characterized by intolerance to cold, especially in extremities, often accompanied by sleep disturbance and fatigue. In addition to heightened sensitivity to cold, individuals with CSC often report symptoms such as poor sleep, shoulder stiffness, and depressive mood, which can collectively reduce their quality of life [4]. However, as CSC is not viewed as a life-threatening condition, standardized diagnostic guidelines, treatment protocols, and preventive strategies are still lacking. According to Takatori, CSC can be diagnosed when a temperature difference of 6°C is detected between the tympanic membrane and the hallux, representing a gap between core and peripheral body temperatures [5]. Using this criterion, we have previously demonstrated a blunted vasodilatory response to endothelial function testing in young women with CSC [6]. At rest, these individuals tend to exhibit reduced parasympathetic nervous activity, contributing to diminished peripheral circulation and skin surface temperature [7]. This suggests that autonomic imbalance may play a key role in the pathophysiology of CSC. On the other hand, footbathing has been shown to promote parasympathetic activity, enhance blood flow to the extremities, and raise skin temperature in healthy individuals, supporting its potential as a therapeutic modality for CSC [8].

In recent years, footbaths have been widely used to improve CSC. However, few studies have examined the effects of footbaths on CSC, and both the types of footbaths and the evaluation indices used vary across studies [9-11]. Therefore, there is no consensus on the effects of footbaths on CSC. Previously, our findings indicated that immersing the feet in warm water led to elevated skin temperatures in both the hands and feet of healthy young women diagnosed with CSC [12]. However, warm-water footbaths can be difficult to prepare because they require large volumes of hot water. In contrast, steam footbaths, which require a small amount of hot water, have also been used in recent years.

Therefore, in this study, we aimed to compare the effects of warm-water and steam footbathing on autonomic nervous activity (ANA indices) and peripheral skin temperature in young women with CSC. Our SMART objectives were: (1) to measure changes in heart rate variability and skin temperature before and after footbathing, (2) to compare these changes between two (warm-water and steam) different types of footbathing interventions (warm-water vs. steam), and (3) to identify which method is more effective within a single 30-minute session.

## Materials and methods

Participants

Healthy female university students from Dokkyo Medical University were invited to participate in this study. Female university students were chosen because CSC is particularly prevalent in women in their twenties, and this population was readily accessible for recruitment within our institution [3]. Those who agreed to join the study did so voluntarily after receiving a full explanation during an in-person campus interview. Compliance with pre-test instructions was verbally confirmed on the day of measurement and noted in the participant checklist.

In women of reproductive age, core body temperature varies across different phases of the menstrual cycle. The normal menstrual cycle duration ranged from 26 to 35 days [13]. Participants reported the dates of their two most recent menstrual periods, and based on this information, we estimated the average cycle duration to confirm it was within a typical range.

The start of menses was determined to be day 1, and days 6-12 were considered the follicular phase [13]. All measurements were scheduled during the follicular phase of the menstrual cycle to minimize hormonal influences on body temperature variability [13]. Therefore, measurements were performed during the follicular phase for each participant. Individuals with a temperature difference of 6°C or more between core and peripheral skin temperature and had subjective symptoms of coldness were defined as having CSC, and were randomly assigned to either the warm-water footbath group or the steam footbath group. To minimize confounding factors, participants were instructed to refrain from consuming alcohol or engaging in vigorous physical activity and to ensure sufficient sleep on the night before testing. In addition, they were required to abstain from food, caffeine, smoking, and any oral intake for at least two hours prior to the scheduled measurements.

Exclusion criteria included pregnancy, flu-like symptoms, chronic disease, or use of medications/supplements. Participants had no history of cerebrovascular or cardiovascular diseases that could affect peripheral circulation, chronic obstructive arterial disease, or any medications or supplements. Individuals engaged in competitive or high-intensity sports were excluded due to their potential influence on autonomic nervous system balance.

This study was conducted from October 2016 to April 2017. In a previous study, we found that over 88% of individuals who experienced cold sensitivity exhibited marked core-to-peripheral temperature disparities during months when the mean ambient temperature exceeded 15°C [14]. Therefore, this study was conducted between autumn and early spring.

Measurement methods

This study is performed in a quasi-experimental, cross-sectional design. No randomization was performed, as this was a cross-sectional comparison study. The test was performed in a room with a temperature of 23.4±2.8°C and a relative humidity of 36.3%±10.8%. Detailed protocols for heart rate variability (HRV) and skin temperature measurement are described below.

A digital thermometer (model CTD505, Citizen Systems, Tokyo, Japan) was employed to assess the tympanic temperature on the right side. Skin surface temperatures at the right big toe and the back of the right hand were simultaneously recorded using an eight-channel wireless thermometer with high measurement precision (Nikki-Thermo, Tokyo, Japan).

Each participant was fitted with chest-mounted radiofrequency ECG sensors (GMS, Tokyo, Japan) for the continuous collection of heart signals. HRV was analyzed in real time using the MemCalc/Bonaly Light system (GMS) and maximum entropy algorithms, based on R-R interval data. Frequency domain analysis was conducted to obtain mean low-frequency (0.04-0.15 Hz) and high-frequency (0.15-0.40 Hz) values per 10-second window. The high-frequency (HF) band reflects vagal modulation, while the low-frequency (LF) band encompasses both sympathetic and parasympathetic input, making the LF/HF ratio a recognized index of sympathetic drive [15]. We used the reference value of HF 975±203 ms; the LF/HF for this ranges from 1.5 to 2.0 [16]. The instruments used for HRV and temperature measurement have been validated in prior studies and are commonly used in physiological research [15,16].

**Figure 1:**
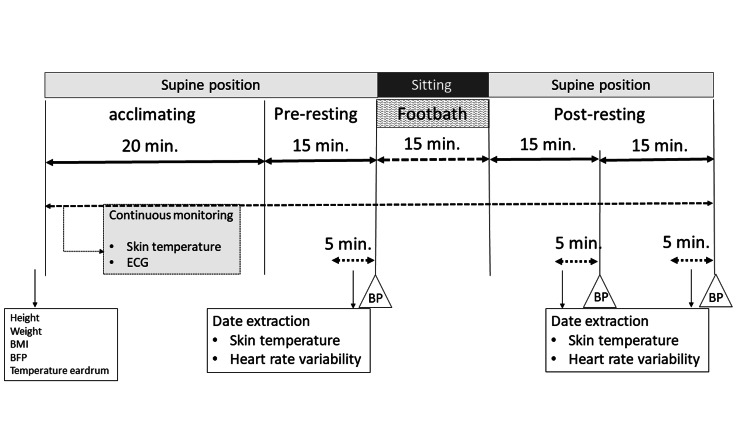
Protocol. Measurements were collected at three time points: 15 minutes before, and 15 and 30 minutes after the footbath session. BP, blood pressure; ECG, electrocardiogram

Participants underwent a 20-minute acclimatization in the supine position, during which skin temperature and cardiac electrical signals were continuously monitored. The last five minutes of the following 15-minute resting phase before the footbath were used as baseline data. Next, participants were seated with back support and immersed their feet in a bath for 15 minutes. A 15-minute duration was selected in accordance with previous studies, which reported it as an effective period for inducing physiological changes through footbathing [9,10]. After the footbath, they returned to the supine posture for 30 minutes, and their bodies were covered with a towel blanket from the neck down to the toes. Measurements were collected at three time points: 15 minutes before and 15 and 30 minutes after the footbath session. Measurements of skin temperature and HRV were collected during the final five minutes of each 15-minute interval post-intervention to reflect stabilized physiological responses (Figure 1). During foot immersion, the skin temperature probe at the toe was removed, and towels were placed over the wrists and knees for insulation.

This investigation adhered to the ethical principles outlined in the Declaration of Helsinki. After providing information about the study, only young women willing to volunteer participated. Ethical clearance was granted by the Institutional Review Board of the School of Nursing, Dokkyo Medical University (approval number: 28009).

Footbath method

The warm-water footbath involved immersing both lower legs into a footbath device (“Hietorikun,” model FB-C80; Koyo Corporation, Sakai, Japan) filled with 10 L of water maintained at 41°C. A portion of each leg was extended from the soles of the feet to a height of 25 cm.

The steam footbath involved placing both lower legs into a steam footbath device (“Steam Foot Spa,” model EH2862P-W; Panasonic, Tokyo, Japan) that produces a constant steam temperature of 42°C, generated by heating 100 mL of water in a designated container. This device heats water electrically, produces steam, and emits it into the footbath chamber through a steam outlet. The device features two modes: “Steam Bath,” which warms the area from the toes to the calves using only steam, and “Far-Infrared Steam Bath,” which additionally warms the calves using far-infrared radiation. Furthermore, two steam intensity modes were available: “Standard,” which gently warms with fluctuating steam emissions, and “Strong,” which provides more rapid and intense warming. The fluctuation of the steam refers to intermittent steam emissions that cause slight variations in the temperature inside the footbath chamber, maintaining a continuous sensation of warmth. The steam temperature could be adjusted across five levels, ranging from approximately 42°C to 46°C.

For this study, in order to compare it with the warm-water footbath, the steam bath mode at the 42°C setting and the “Steam Bath” and “Standard” steam modes were selected. The steam device maintained a constant temperature of 42°C throughout the footbathing session in standard mode.

**Figure 2:**
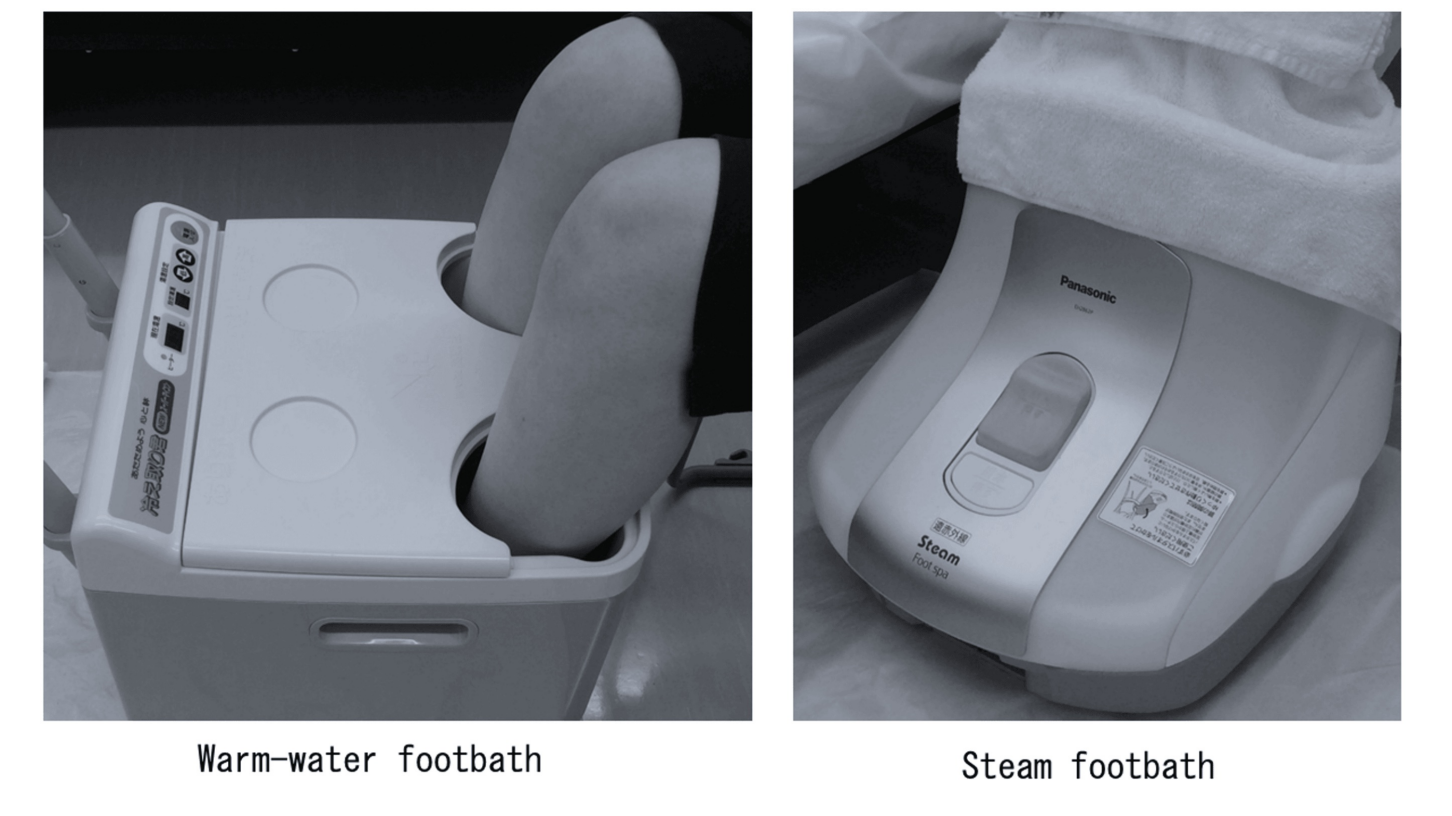
Photographs of warm-water footbath and steam footbath

The steam footbath chamber covered up to 10 cm below the patella and to prevent steam from escaping from the top of the footbath, a bath towel was placed over the area from the top edge of the chamber to the portion covering the knees, sealing any gaps. Both types of footbaths were performed for 15 minutes per session, with the participant seated in a chair (Figure 2).

Participants were randomly assigned to either the warm-water foot bath group or the steam foot bath group by placing papers with the two types of characters written on them into envelopes and drawing lots.

Statistical analysis

The most stable five-minute data of the obtained measurements (excluding tympanic temperature and blood pressure) were used. The change after the footbath with respect to the pre-footbath status is expressed as the mean±standard error. Baseline data and data collected at 15 and 30 minutes after the footbath were compared. The Kolmogorov-Smirnov test with Lilliefors correction was used to evaluate normality. Depending on the distribution, either t-tests or Wilcoxon signed-rank tests were used. The statistical methods employed are standard in clinical and physiological research, especially for small sample studies with normally distributed data [6]. A significance threshold was set at p<0.05. All statistical analyses were conducted using IBM SPSS Statistics version 19.0J for Windows (IBM Corp., Armonk, NY).

## Results

Basic characteristics

**Figure 3:**
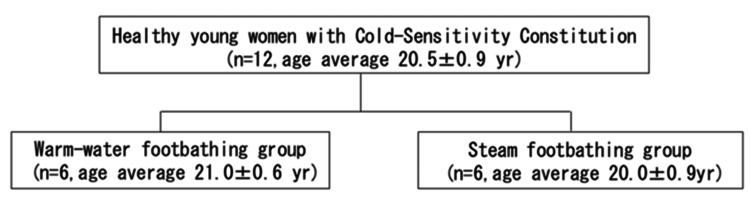
Diagram of the classification of study participants into groups

Overall, 12 healthy young women, with a mean age of 20.5±0.9 (range, 18‒23) years, completed the study. Six participants were in the warm-water footbathing group (mean age, 21.0±0.6 years) and six in the steam footbathing group (mean age, 20.0±0.9 years) (Figure 3).

Physical characteristics of the participants are presented in Table 1. Age, height, weight, body mass index, body fat percentage, right tympanic membrane temperature, right big toe skin temperature at rest, smoking, drinking, and exercise habits were not significantly different between the two (warm-water and steam) groups.

**Table 1:** Comparison of baseline characteristics between warm-water footbathing group and steam footbathing group Data are expressed as mean±SD. BMI, body mass index; BFP, body fat percentage. Statistical significance was defined as p<0.05.

	Warm-water footbathing group (n=6)	Steam footbathing group (n=6)	p-value
Age (years)	21.0±0.6	20.0±0.9	0.026
Height (cm)	156.6±3.7	158.8±4.1	0.699
Weight (kg)	50.3±3.7	53.3±3.0	0.240
BMI (kg/m^2^)	20.5±0.8	21.1±1.2	0.589
BFP (％)	32.2±2.3	30.2±3.0	0.240
Temperature eardrum (˚C)	36.1±0.4	36.1±0.4	0.937
Temperature hallux (˚C)	24.5±1.9	23.1±3.8	0.310
Current smoking: n (%)	0 (0)	0 (0)	-
Drinking: n (%)	1 (16.7)	0 (0)	0.296
Exercise habit: n (%)	1 (16.7)	1 (16.7)	1

Changes in heart rate, blood pressure, and HRV

**Figure 4:**
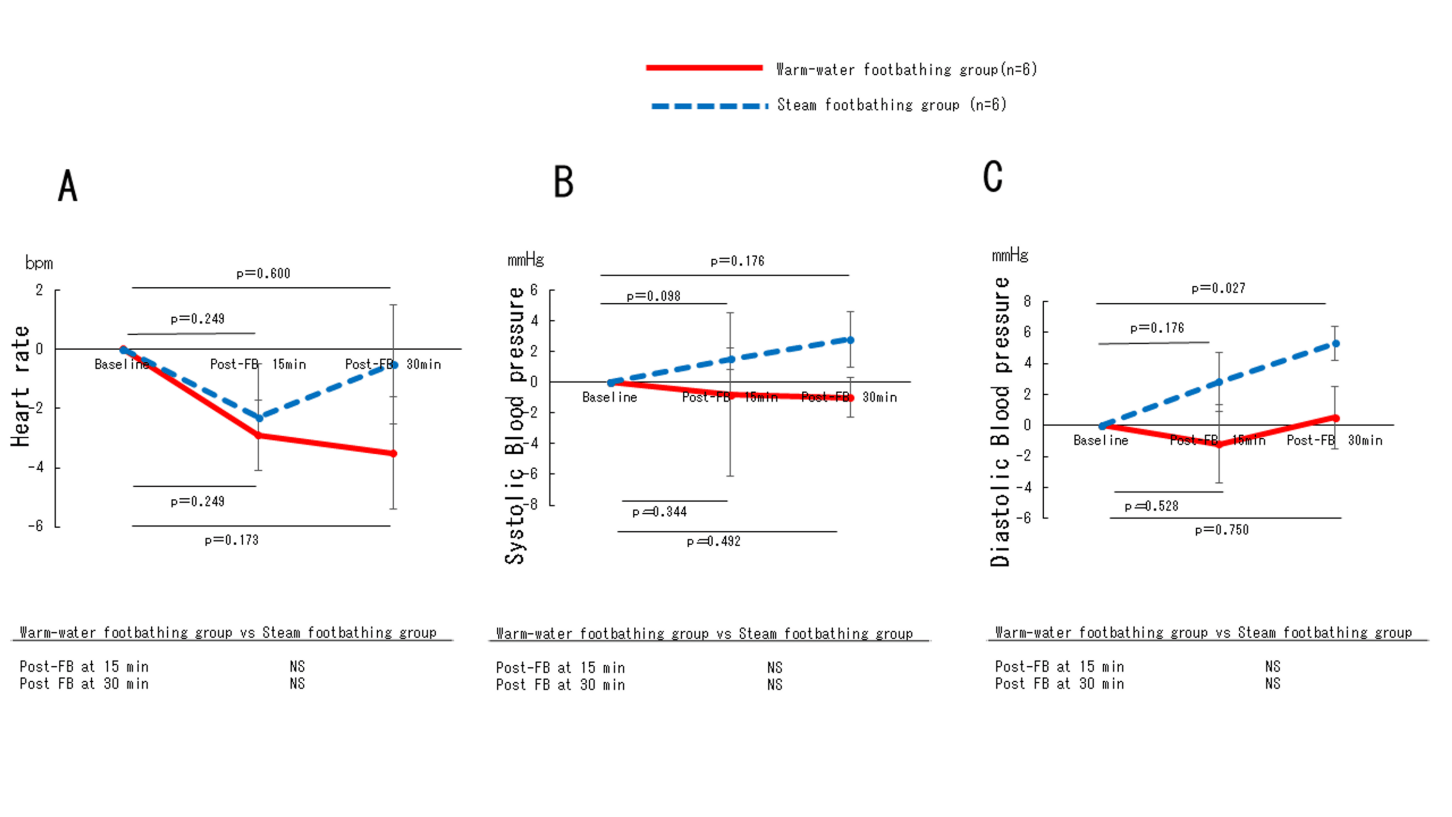
Changes in heart rate (A) and systolic blood pressure (B) and diastolic blood pressure(C) during test periods. Data are presented as mean±SE. Asterisks indicate significant differences compared to baseline: *p<0.05, *p<0.01. Statistical significance was set at p<0.05. FB, footbath; min, minute: NS, not significant.

**Figure 5:**
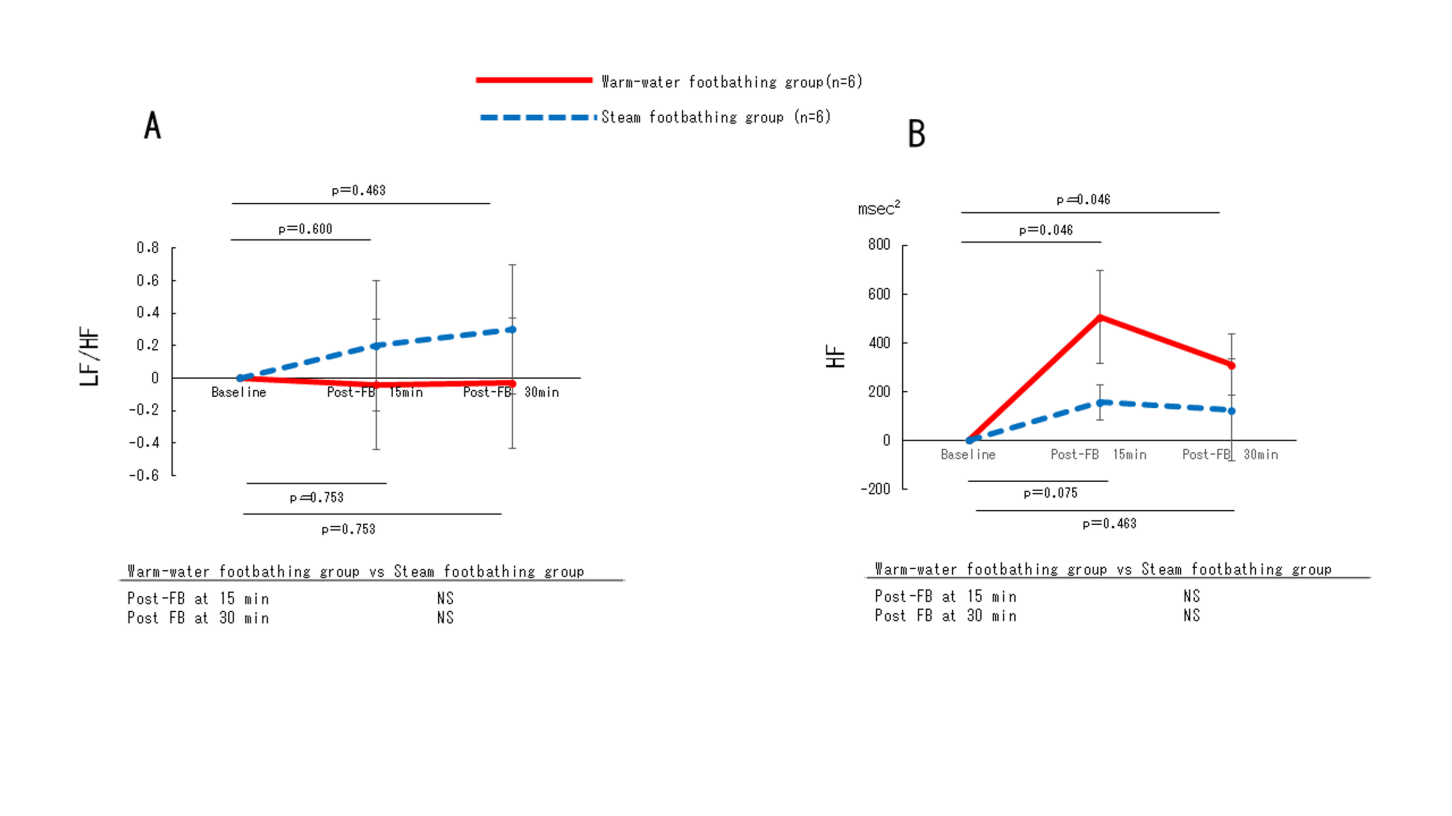
Changes in LF/HF (A) and HF (B) during test periods. Data are presented as mean±SE. Asterisks indicate significant differences compared to baseline: *p<0.05, *p<0.01. Statistical significance was set at p<0.05. LF, low-frequency; HF, high-frequency; FB, footbath; min, minute: NS, not significant.

Heart rate (Figure 4A), systolic blood pressure (Figure 4B), and diastolic blood pressure (Figure 4C), and LF/HF (Figure 5A) were not significantly different between the two (warm-water and steam) groups. The HF (Figure 5B) showed a more increasing trend (p=0.065) in the warm-water footbathing group than in the steam footbathing group, although this was not statistically significant after 15 minutes of footbathing (Table 2).

**Table 2:** Changes in clinical variables at 15 minutes and 30 minutes post-footbathing (FB) between warm-water footbathing group and steam footbathing group Data are expressed as mean±SE. Group comparisons were made at each time point. Statistical significance was defined as p<0.05.LF, low-frequency; HF, high-frequency.

	Post-FB 15 min			Post-FB 30 min		
	Warm-water footbathing group	Steam footbathing group	p-value	Warm-water footbathing group	Steam footbathing group	p-value
Heart rate (bpm）	-2.9±1.2	-2.3±1.8	0.818	-3.5±1.9	-0.5±2.0	0.485
HF (msec^2^）	505.2±190.7	155.6±72.4	0.065	310.1±124.4	123.3±208.2	0.699
LF/HF	-0.04±0.4	-0.2±0.4	1.000	-0.03±0.4	0.3±0.4	0.310
Systolic blood pressure (mmHg）	-0.8±5.3	1.5±0.7	0.093	-1.0±1.3	2.8±1.8	0.132
Diastolic blood pressure (mmHg）	-1.2±2.5	2.8±1.9	0.240	0.5±2.0	5.3±1.1	0.065
Temperature dorsal hand (℃）	0.6±0.5	-0.3±0.4	0.930	0.8±0.4	-0.6±0.5	0.065
Temperature hallux (℃）	6.3±1.6	7.0±1.3	1.000	9.0±0.6	5.7±1.0	0.026

Regarding temporal changes within each group, diastolic blood pressure increased significantly in the steam footbathing group after 30 minutes of footbathing (p=0.027), and HF increased significantly in the warm-water footbathing group after 15 and 30 minutes of footbathing (p=0.046 at both 15 and 30 minutes).

Changes in body temperature and cutaneous blood flow

**Figure 6:**
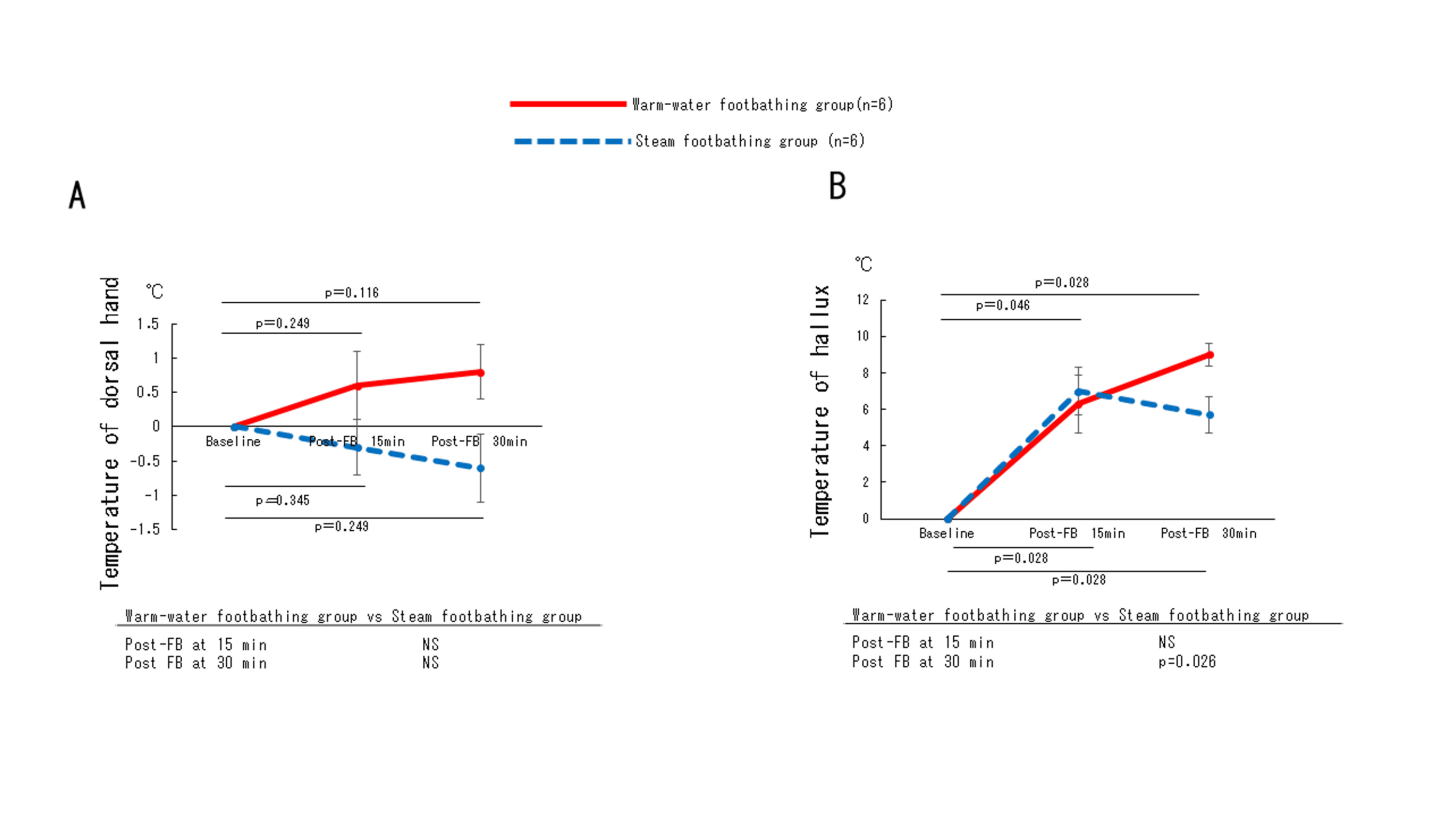
Changes in temperature of dorsalhand (A) and hallux (B) during test periods. Data are presented as mean±SE. Asterisks indicate significant differences compared to baseline: *p<0.05, *p<0.01. Statistical significance was set at p<0.05.FB, footbath; min, minute: NS, not significant

The increase in dorsal hand skin temperature (Figure 6A) was not significantly different between the warm-water and steam footbathing groups at 15 and 30 minutes after footbathing. Toe skin temperature (Figure 6B) showed a significant increase in the warm-water footbathing group compared to the steam footbathing group at 30 minutes (p=0.026).

The toe skin temperature increased significantly in both the warm-water and steam footbathing groups at 15 and 30 minutes after the footbath (p=0.046 at 15 minutes for warm-water footbathing; p=0.028 for steam footbathing; p=0.028 at 30 minutes for both warm-water and steam footbathing).​​​​​

## Discussion

This study specifically focused on young women with CSC, a population underrepresented in previous research. Our findings provide early evidence for selecting appropriate footbath methods in this demographic.

In this study, young women in their twenties with self-reported cold sensitivity and a core-to-peripheral temperature difference of ≥6°C were evaluated to compare the effects of warm-water and steam footbathing on autonomic nervous activity and peripheral skin temperature. Participants underwent footbathing under controlled conditions, and physiological changes were monitored at baseline and at 15 and 30 minutes post-intervention.

Our findings demonstrated that HF, an index of parasympathetic nervous activity, tended to increase following warm-water footbathing, while this effect was not observed in the steam group. Although this difference did not reach statistical significance, the within-group analysis showed a significant increase in HF at both 15 and 30 minutes after footbathing in the warm-water group. These findings are consistent with those of Kaneko et al. [8], who reported enhanced parasympathetic activity following warm thermal stimulation. This suggests that warm-water footbathing may be beneficial in shifting autonomic balance toward parasympathetic dominance in individuals with CSC.

The LF/HF ratio, which reflects sympathetic modulation, showed no significant changes in either group. This aligns with previous studies showing that footbathing primarily affects the parasympathetic branch of the autonomic nervous system, with limited impact on sympathetic activation [10].

Regarding peripheral skin temperature, the hallux skin temperature increased significantly in the warm-water group compared to the steam group at 30 minutes post-footbathing. In contrast, dorsal hand temperature showed an increasing trend, but no statistically significant differences were observed. Our findings support previous work by Ogata et al. [7] and Funato et al. [11], who demonstrated improvements in peripheral circulation following thermal stimulation of the lower extremities. The stronger thermal effect in the warm-water group may be attributed to the larger volume of hot water, greater immersion depth, and more consistent temperature distribution compared to the steam method. The first is the difference between the amount of hot water used and the method of thermal stimulation of the feet. In steam-type footbathing, approximately 100 mL of water was used to warm the feet by using an intermittent steam jet. Warm-water footbathing used approximately 10 L of hot water, which is approximately 100 times more than in steam footbathing, and the lower legs were immersed in hot water, which kept them at a constant temperature. The warm-water immersion covered more of the lower leg (25 cm) compared to the steam footbath (10 cm below the patella), potentially contributing to differences in thermal stimulus.

Although CSC (Hiesho) is culturally rooted in East Asia, its physiological basis shares features with the internationally recognized concept of cold intolerance [17,18]. The temperature-based diagnostic approach applied in this study may provide a cross-cultural framework for future comparative research.

From a physiological perspective, thermal stimulation of the plantar surface - where a high density of thermoreceptors is present - may activate sensory input to the hypothalamus, leading to increased parasympathetic tone and peripheral vasodilation [19]. Our study supports this mechanism, as greater changes in HF and skin temperature were observed with warm-water footbathing.

Importantly, this study focused specifically on healthy young women, a group underrepresented in prior studies, which often included mixed-age samples or menopausal women [9]. By controlling for age, menstrual phase, and health status, we were able to isolate the physiological effects of the intervention.

Nevertheless, several limitations should be acknowledged. First, the small sample size limits statistical power and generalizability. However, this pilot study serves as a foundation for future research with larger cohorts. Second, although we measured objective physiological parameters, subjective outcomes such as perceived warmth and comfort were not assessed. Third, the steam footbath device used had limited temperature customization, which may have influenced the results.

In conclusion, warm-water footbathing may be a more effective non-invasive intervention than steam footbathing for increasing parasympathetic activity and peripheral circulation in young women with CSC. Further studies are warranted to confirm these findings and explore long-term effects.

## Conclusions

The study involved 12 healthy young women with self-reported cold sensitivity and a ≥6°C difference between right tympanic and right great toe temperatures. Participants were divided into two (warm-water and steam) groups: a warm-water footbath group (n=6) and a steam footbath group (n=6). Heart rate variability, skin temperature, heart rate, and blood pressure were measured. The results showed that the parasympathetic activity index tended to increase in the warm-water footbathing group 15 minutes after footbathing, although the difference was not statistically significant. No statistically significant differences were observed in sympathetic nerve activity index or heart rate between the two (warm-water and steam) groups. The right dorsal hand skin temperature showed an increasing trend in the warm-water footbathing group at 15 and 30 minutes after the footbath, although the difference was not statistically significant. Notably, the right hallux skin temperature increased significantly more in the warm-water footbathing group 30 minutes after footbathing. Warm-water footbathing for healthy young women with CSC increased PNA and peripheral skin temperature more effectively than steam footbathing. These conclusions are based on the current sample and methodology and should be interpreted within the context of this pilot study.
